# Pediatric Dental Emergency Visits and Treatment During Lockdown in the COVID-19 Pandemic: A Retrospective Analysis at the Pediatric Emergency Unit of the University Clinic of Dentistry, Vienna, Austria

**DOI:** 10.3390/jcm14072359

**Published:** 2025-03-29

**Authors:** Ali Al Ibraheem, Sophie Lembacher, Laura Urban, Katrin Bekes

**Affiliations:** Department of Paediatric Dentistry, University Clinic of Dentistry, Medical University Vienna, Sensengasse 2a, 1090 Vienna, Austria; all.alibraheem@meduniwien.ac.at (A.A.I.);

**Keywords:** COVID-19, pandemic, lockdown, pediatric dentistry, emergency visit

## Abstract

**Background**: In the beginning of the COVID-19 pandemic, the Department of Pediatric Dentistry at the University Dental Clinic Vienna switched from routine medical care to emergency operation mode. The study aims to retrospectively compare the characteristics of pediatric patients’ emergency visits before, during, and after lockdown. **Methods**: All pediatric emergencies that presented at the Department of Pediatric Dentistry in the period from the 7 January 2020 to the 31 July 2020 were recorded. Patients were subclassified into three groups with regard to their time of presentation (group 1: before lockdown, group 2: during lockdown, group 3: after lockdown). Then, pediatric patients’ purposes for emergency visit, diagnoses, and the treatments practiced were compared. **Results**: The number of patients was significantly lower during lockdown but increased again afterward. During lockdown, significantly more patients presented with dental pain, the diagnosis “Extraction: tooth not restorable” increased, and extractions were significantly more often the treatment of choice (*p* < 0.05). **Conclusions**: The presented study is the first research on pediatric dental emergency visits during the first lockdown of the COVID-19 pandemic in Austria. The results highlight the negative impact of the pandemic on pediatric dental care as the number of treatments decreased, elective treatments were postponed, and the diagnosis and prognosis of teeth deteriorated.

## 1. Introduction

At the beginning of 2020, the World Health Organization (WHO) confirmed that a novel coronavirus causing a respiratory disease was first detected in Wuhan, Hubei, China. From there on, the virus spread globally within few months, resulting in a pandemic disease [1]. In March, the Austrian government declared a national emergency in response to escalating cases of coronavirus infections. Eventually, the continuous spread culminated in nationwide restrictive measures starting from the 16 March 2020. The Austrian government ordered a lockdown, which greatly changed protocols in health care institutions including dental facilities. Dentists were faced with a particularly high risk of being exposed to SARS-CoV-2, as a safe interpersonal minimum distance cannot be maintained during treatment. Additionally, the exposition to aerosol and droplets generated by many dental procedures poses a higher risk of airborne infections, especially through asymptomatic patients [2,3]. Therefore, appropriate conscientious and protectional measures were required [4,5,6]. Dental professionals were instructed to adapt their daily routine and install rigorous safety protocols to minimize the risk of infection for health care workers and patients [7]. In accordance with public health guidelines of the Austrian Ministry for Health, the University Dental Clinic Vienna switched from routine medical care to emergency operation mode. To stop the virus from spreading further, all general non-emergency treatments were suspended, and elective dental procedures were postponed [8]. The treatment protocol was strictly limited to dental emergencies. Children experiencing oral pain or severe dental complications requiring urgent examination were classified as emergency cases and admitted accordingly. However, patients with fever or other COVID-19-related symptoms were not granted access. Upon arrival, all patients underwent an initial screening for COVID-19 symptoms, including body temperature measurement, assessment of breathing difficulties, and sore throat evaluation. Only those who passed the screening were admitted. Following admission, strict safety protocols were enforced, including the use of sanitizers, gloves, face masks, and adherence to social distancing measures. The study aims to analyze patient flow and characteristics based on the purpose of their dental visit, diagnosis, and treatment across three periods—before (7 January 2020–13 March 2020), during (16 March 2020–15 May 2020), and after lockdown (18 May 2020–31 July 2020)—at the Department of Pediatric Dentistry, Medical University of Vienna. Additionally, it seeks to investigate the epidemiological impact of the COVID-19 pandemic on these patterns.

## 2. Methods

The retrospective analysis includes all children aged 0 to 15 years, as 15 is the maximum age for admission to the pediatric department. It examines cases presented at the Emergency Unit of the Department of Pediatric Dentistry, University Clinic of Dentistry, Medical University of Vienna, Austria, between 7 January 2020, and 31 July 2020 (covering periods before, during, and after lockdown, with almost 10 weeks for each period). To account for the duration of the first lockdown, the retrospective data analysis begins on the first working day of 2020, extends through the lockdown period, and concludes with a post-lockdown phase matching the duration of the lockdown itself.

Therefore, patients were subclassified into three groups with regard to their time of presentation at the pediatric emergency department:

- Group 1: patients who presented before the start of the COVID-19 pandemic:

t(0): 7 January 2020 to 13 March 2020.

- Group 2: patients who presented during the first lockdown:

t(1): 16 March 2020 to 15 May 2020.

- Group 3: patients who presented immediately after the first lockdown:

t(2): 18 March 2020 to 31 July 2020.

Patients were identified using the Med-Folio electronic record card system of the University Clinic of Dentistry. Relevant computerized data were extracted from the plain text of electronic patient records which are documented by each dentist individually. The documentation covers the initial general impression of the patient, general medical history, social and dental anamnesis, dental status, diagnosis, and treatment plan. Relevant data such as gender, age, reason for consultation, diagnosis, and therapeutic measures were filtered from each patient and pseudonymisedly recorded in an Excel table with MS Excel 14.0.0. The study aims to analyze patient flow and characteristics based on the purpose of their dental visit, diagnosis, and treatment across three periods—before (7 January 2020–13 March 2020), during (16 March 2020–15 May 2020), and after lockdown (18 May 2020–31 July 2020)—at the Department of Pediatric Dentistry, Medical University of Vienna. It also aims to compare the different reasons for admission and explore potential treatment approaches during these three periods. Additionally, the study seeks to investigate the epidemiological impact of the COVID-19 pandemic on these patterns.

Statistical analysis was performed using the IBM SPSS Statistics program (IBM Corp., IBM SPSS Statistics for Windows, Version 29.0.2.0, Armonk, NY, USA) on the basis of data which had previously been anonymized in Excel tables. Descriptive statistics (mean, SD) were provided for continuous measurements (age). Nominal measurements (e.g., gender, type of diagnosis, type of treatment) are summarized using frequencies and proportions as well as crosstabulations. For the inferential statistics, a chi-square test compared the purpose of the visits, diagnosis, and treatment procedures before, during, and after lockdown periods by assuming significance at *p* ≤ 0.05.

## 3. Results

### 3.1. Demographic Characteristics

**Figure 1:**
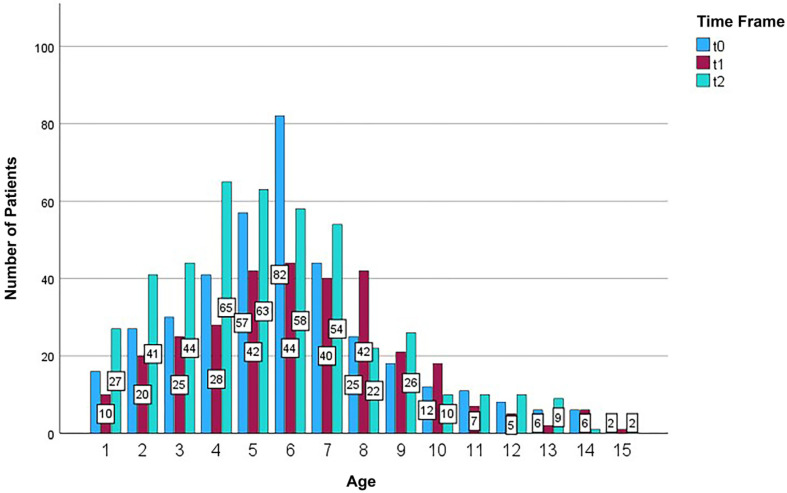
Patient demographics: Age distribution before (t_0_), during (t_1_), and after lockdown (t_2_).

Before lockdown, 385 patients sought medical consultation. During lockdown, patient numbers fell to a total of 311 children, only to rise again to 442 children after lockdown. In total, the sample comprised 1138 patients who presented at the Emergency Unit of the Department of Pediatric Dentistry between 7 January 2020 and 31 July 2020. The mean age was 5.89 years (SD 2.91 years), range: 1–15 years). A peak was observed between the ages of three and seven years (*n* = 717) (Figure 1). At 52% (*n* = 592), the majority of study participants were boys; 48% (*n* = 546) were girls. While 187 boys presented before lockdown, only 150 presented during lockdown, and 255 after lockdown. With 198 patients presenting before, 161 during, and 187 after lockdown, no relevant fluctuation was observed among girls.

### 3.2. Patient Numbers

**Figure 2:**
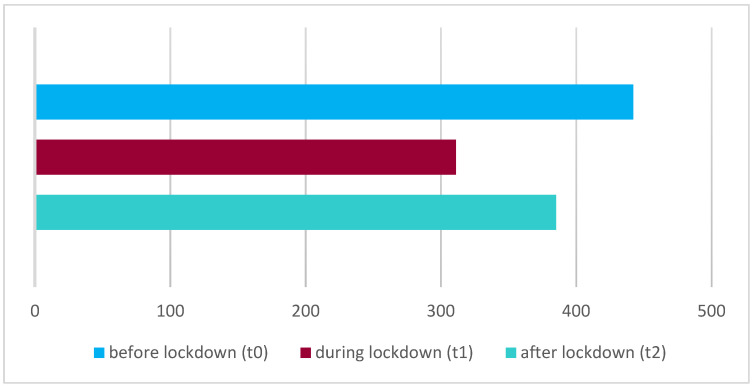
Number of patients before (t_0_), during (t_1_), and after lockdown (t_2_).

A look at the fluctuation of patient inquiries reveals the correlation between patient numbers and the time of lockdown. Before lockdown, 385 patients sought medical consultation. During lockdown, patient numbers fell to a total of 311 children, to then only rise again to 442 children after lockdown. There is a statistically significant relationship between the number of patients and the three time periods (before, during, and after the lockdown) (Figure 2). For this analysis, t_2_ was compared with t_0_, and t_1_ was compared with t_0_. This allowed for a separate assessment of whether the number of patients was statistically significantly higher after the lockdown than before. Additionally, it was examined whether the number of patients was statistically significantly lower during the lockdown compared to before. The chi-square test indicates that the absolute number of presentations during the lockdown was significantly lower (*p* < 0.005); after the lockdown, it increased significantly (*p* = 0.047).

### 3.3. Purpose of Visits

Analyzing the motive for presentation throughout all observed time periods, the most prevalent reason for consultation was dental pain (total: 47.7%, *n* = 543, t_0_: 37.4%, *n* = 144, t_1_: 54.7%, *n* = 170; t_2_: 51.8%, *n* = 229), followed by patients either seeking a routine check-up or a second opinion (total: 12.6%). Of the total, 11.7% (*n* = 133) of patients presented with traumatic dental injury, and 3.3% (*n* = 38) of patients sough help because of tooth-related infections. Seventy-six patients were referred to the Emergency Unit of the Department of Pediatric Dentistry (6.7%). The separate analysis for the respective time periods (t_0_, t_1_, t_2_) shows that before lockdown, 37.4% of patients presented with dental pain (*n* = 144). During lockdown, the share increased to 54.7% (*n* = 170). After lockdown, the share of patients seeking help for dental pain increased to 73.6% (*n* = 229). Chi-square statistical tests established that during lockdown, there were significantly more patients presenting with pain in comparison to before lockdown (*p* < 0.05). Table 1 gives an overview of the different motives for presenting at the Pediatric Emergency Department. It is also worth mentioning that the “Routine Check-up/Follow-up” category includes patients who required a standard check-up, both before and after the lockdown period, as well as those who needed follow-up care during the lockdown for various reasons. For instance, this could include patients who visited the emergency clinic during lockdown and required a course of antibiotics before undergoing certain procedures, such as extractions. Additionally, it covers patients who needed post-treatment monitoring, such as suture removal or the completion of ongoing endodontic treatments. The “Referral” category includes patients who were previously seen by general practitioners or dentists from other specialties but were referred due to a lack of expertise in treating children or performing the necessary procedures. As a result, these patients were admitted under the “Referral” classification. The “Need for Orthodontic Treatment” category applies exclusively to patients seen before and after the lockdown. As shown in Table 1, no patients in this category were received during the lockdown period.

### 3.4. Diagnosis

At 43.5% (*n* = 495), the most prevalent diagnosis throughout all three time periods was “caries/insufficient fillings”, followed by “teeth not worthy of preservation/extraction/orthodontic tooth extraction” (42.4%, *n* = 483). The separate analysis for the respective time periods (t_0_, t_1_, t_2_) shows that in absolute numbers, the number of patients with a tooth not worthy of preservation continuously rose from 138 patients before lockdown to 158 patients during lockdown, and to 187 patients after lockdown. In relation to the total number of patients presenting at the Emergency Unit of the Department of Pediatric Dentistry, the relative share of patients diagnosed with a tooth not worthy of preservation initially increased from 35.8% of patients before lockdown to 50.8% during lockdown. A chi-square test confirmed that the diagnosis “Extraction” was made significantly more often during lockdown compared to the period before lockdown (*p* < 0.05). However, after lockdown, the share only amounted to 42.3%, and no statistically significant differences were found between the number of diagnoses of “tooth not worth saving” in the time periods “before lockdown” (t_0_) and “after lockdown” (t_2_). Table 2 summarizes the different diagnoses before, during, and after lockdown.

### 3.5. Treatment Procedures

In total, in 179 of 1138 patients, teeth were extracted (15.7%). Before lockdown, tooth removal was the chosen form of therapy in 13.8% (*n* = 53) of cases; during lockdown, the number rose to 23.5% (*n* = 73), to only reduce again to 12.0% (*n* = 53) after lockdown. Compared to the time before lockdown, statistically significantly more extractions were performed during lockdown (*p* < 0.05). However, there were no significant differences in the number of “extraction” therapy measures before and after lockdown (*p* = 0.447). Of all patients, 959 received different forms of therapy other than the extraction of a tooth. A total of 86.2% (*n* = 332) of these alternative therapeutic measures were conducted before lockdown, 76.5% (*n* = 238) during, and 88.0% (*n* = 389) after lockdown. The most prevalent therapy recommendation was directing patients towards becoming a regular patient of the department in order to establish a detailed treatment plan and enable a sustainable full-mouth rehabilitation (40.9%). The number of recommendations towards treatment under general anesthesia tripled from 45 patients (11.7%) before lockdown to 123 patients (27.8%) after lockdown. A total of 27.7% (*n* = 315) of cases were either referred to other departments within in the University Dental Clinic or to extramural residential dentists. A total of 26.6% (*n* = 303) of patients were prescribed adequate medication. Table 3 gives an overview of the different treatment procedures performed at the Emergency Unit of the Department of Pediatric Dentistry before, during, and after lockdown.

## 4. Discussion

This study examined the purpose of dental clinic visits, diagnoses, and treatments of pediatric patients during the lockdown period. The aim was to analyze the effects of a global health crisis on emergency dental treatment in pediatric dentistry in Austria. Furthermore, the study sought to provide information on how to improve crisis management in the future by adapting operation protocols.

The methodic approach of subgrouping the patient collective into three chronological entities (before, during, and after lockdown) allowed a sufficient analysis of possible lockdown-associated changes in the modus operandi of emergency treatment in pediatric dentistry. Previous studies have opted for a similar approach [9,10]. In addition, the evaluation of retrospective data allowed the observation of a more extensive period of time and the identification of specific characteristics of the different phases of the pandemic [11].

While the development of age and gender distribution was similar throughout all three phases (t_0_, t_1_, t_2_), the results show that during lockdown, the number of patients visiting the emergency unit significantly decreased (*p* = 0.005). Similar studies in China [12], Turkey [13], and Israel [4,10] confirmed a reduction in patient numbers. The dramatic drop in patient numbers in their respected emergency units, especially in the early stages of the pandemic, show the significant impact the lockdown had on dental care [14]. While patients quickly adapted to the newly imposed restrictions during the lockdown, patients’ decisions to take up health services was yet greatly influenced by fear and insecurity. Studies show that people were eager to reduce the workload of health workers, feared a higher risk of infection, and were unsure how to correctly act upon the information given by government and other channels. In consequence, many caregivers avoided or postponed necessary dental treatment [7,15]. As schools were closed and sport and other leisure activities were greatly limited, fewer traumatic injuries were expected. The number of dental traumatic injuries dropped from 45 (t_0_) to 26 (t_1_) patients during lockdown. On the other hand, an increase in the number of patients seeking emergency treatment was expected after the lockdown compared to the time before lockdown. As many dental offices were entirely closed or limited their opening hours, the availability of dental services during lockdown was severely impacted. In Austria, 72.4% of pediatric dentists had their practices open; 78.6% only offered emergency services. No dentist offered services on a regular basis to a full extent [8]. This may have made it difficult for caregivers to find an appropriate appointment [8,16] during the lockdown. As many households were faced with loss of income, financial considerations may have also attributed to the decline in medical consultation. The results are worrying, as a lack of pediatric dental care negatively impacts general health and wellbeing [17]. Untreated dental problems can cause dental pain, infections, and long-lasting health impairments [18,19,20].

In accordance with previous studies, the relative share of patients presenting with dental pain significantly increased during lockdown (*p* = 0.000005) [4,10,13]. Madi et al. [21] also confirm these results on the demand of dental emergency treatment during the pandemic. They also observed a significant drop in patient numbers during the pandemic and showed that patients’ visits due to acute dental pain significantly increased. Two factors are considered relevant in interpreting these results. Firstly, caregivers seemed rather reserved to consult health professionals, as they sought to minimize the risk of infection with SARS-CoV-2 in health institutions. Additionally, there was the aforementioned limited availability of dental care (reduced office hours or complete shutdown of dental offices). Therefore, patients only seemed to take up professional help in utmost emergencies, such as severe dental pain [22], therefore, resulting in an increase of patients presenting with dental pain during the pandemic. As dental pain usually calls for immediate action to relieve the pain, health professionals are consulted. It is possible that the increased number of patients with dental pain stems from the previous postponement of necessary treatment due to fear of infection in health institutions. Studies have shown that dental pain can cause a severe deterioration of oral health-related quality of life [23,24]. Ignoring or suppressing pain can provoke further complications and compromise or delay recovery later. This highlights the effects of the pandemic on oral health and emphasizes the importance of professional care and support, especially in vulnerable times.

Moreover, significantly more children were diagnosed with a tooth not worth saving (*p* = 0.000072) during lockdown than before lockdown. The relative share increased from 35.8% to 50.8%. Naturally, the share of extractions also increased significantly from 13.8% to 23.5%. The highest number of extractions of all three periods was recorded during lockdown (*n* = 73). Related studies support the observation of a rise in tooth extractions during the pandemic [4,5,13]. Nijakowski et al. [25] also noticed a significant shift in the spectrum of therapeutic measures during the pandemic. In cases where teeth were considered infected or not restorable, the extractions were not always performed. Compliance in children varies, and treatments cannot always be carried out as planned. Additionally, patients do not always attend their scheduled appointments, often seeking help elsewhere or postponing treatment if they no longer experience pain or acute symptoms after taking the prescribed medication. These factors contribute to the varying ratios in our results.

Conservative procedures such as fillings or root canal treatments were significantly less practiced, while the number of surgical interventions increased, in spring 2020. In general, the number of emergency and invasive surgical treatment rose significantly, whereas the number of planned and scheduled conservative treatment approaches decreased [25]. Therefore, the pandemic heavily influenced the spectrum of therapeutic measures, especially in the acute phase of lockdown. As local dental offices were partially closed or limited their office hours, patients with the diagnosis of a tooth not worth saving chose to present at the Emergency Unit of the Pediatric Dentistry Department to proceed with the indicated treatment of tooth extraction. Moreover, the results suggest that the shift to emergency operation mode, in combination with the uncertainty of follow-up treatment options, enhanced a radical treatment approach leaning towards tooth extraction. By postponing dental therapy in the beginning of the pandemic, the prognosis of tooth preservation deteriorated [11,22]. The number of children needing general anesthesia increased from 45 (11.7%) before lockdown to 123 (27.8%) after the lockdown. The results emphasize the requirement of proper pediatric dental care and the promotion of oral health education among caregivers and children [12].

Finally, this study’s findings should be framed by acknowledging its strengths and limitations. The points of measurement were deliberately chosen, to gather a sufficient overview and enable interesting comparison models (before, during, and after lockdown). This provided the grounds for a sound estimation on how the lockdown impacted the pediatric dental care during the COVID-19 crisis. While the study design allowed for the analysis of a larger patient collective, not all records were complete due to the retrospective approach. Upholding basic medical care in the Emergency Unit of the Department of Pediatric Dentistry in times of crisis appeared to be at the expense of detailed documentation. Incomplete records reduced the quantity and quality of the presented data. With limited resources in regard to time and personnel, the main focus was, however, the best possible treatment of patients. Nonetheless, the implementation of internal strategies to improve standardized documentation also in times of crisis is strongly advised. Furthermore, a multicenter approach with the generalized installation of standardized documentation protocols is recommended in order to gain a more comprehensive overview on the impact of the lockdown on pediatric dental care in Austria, explore regional differences, and improve the efficacy of pediatric dentistry for critical future events. As this study mainly focuses on pediatric patients’ visits due to the emergency unit in different periods, further studies must fill the study gaps related to the adult population and non-emergency cases. The examination of further possibly influential variables such as changes in eating and oral hygiene habits, higher stress and fear levels, limitations in the availability of professional oral cleaning, and routine controls is advised, as previous studies have suggested a correlation between eating habits [26], high stress levels [27], fear [28], and poor oral health. The influence of these additional factors poses an interesting angle for future studies. On the whole, such studies will contribute to the scientific literature facilitating the preparation and management of challenging situations in the future. The fear of leaving the safety of one’s home during lockdown, resulting in a decline of dental consultations for children, demands better crisis management in the future to ensure adequate medical care for children in times of crisis. This entails the promotion of educational campaigns on safety measures in dental offices, the timely provision of safety masks and clothing, and standardized strategic action plans for future pandemics. Lastly, a possible bias in reference to the number of patients and the time period must be acknowledged. In that regard, the comparison of the presented data with the data of previous years based on the same time frame is considered helpful.

## 5. Conclusions

To our knowledge, the presented study is the first research on pediatric dental emergency visits during the first lockdown of the COVID-19 pandemic in Austria. The results correlate with previously published international data and emphasize the negative impact that the pandemic had on pediatric dental care. The number of treatments significantly decreased, elective treatments were postponed, and the diagnosis and prognosis of teeth deteriorated. Therefore, the study highlights the importance of a structured approach to allow strategic preparation for future challenges of pandemic scenarios. To avoid similar outcomes in the future, the improvement of crisis management tools is strongly advised. The promotion of educational campaigns on oral health in children, the thorough revision of emergency concepts, including an improvement of the local distribution and accessibility of safety gear for dental offices, and socioeconomic support for low-income families is favorable. The study documents the impacts of an extremely rare global health crisis and encourages further research to support a better understanding of pediatric dental care during the pandemic.

## Figures and Tables

**Table 1:** Purpose of pediatric emergency visits before (t_0_), during (t_1_) and after lockdown (t_2_), significant results are highlighted.

Purpose of Visit		t_0_ Before Lockdown	t_1_ During Lockdown	t_2_ After Lockdown	Total
routine check-up/follow-up	N	72	20	51	143
	%	18.7	6.4	16.4	12.6
dental trauma	N	45	26	62	133
	%	11.4	8.4	19.9	11.7
infection	N	22	10	6	38
	%	5.7	3.2	1.9	3.3
dental pain	N	144	170	229	543
	%	37.4	54.7	73.6	47.7
need for orthodontic treatment	N	1	0	4	5
	%	0.3	0.0	0.9	0.4
referral	N	43	8	25	76
	%	11.2	2.6	8.0	6.7
other	N	74	84	72	230
	%	19.2	27.0	23.2	20.2

**Table 2:** Diagnosis among pediatric patients before (t_0_), during (t_1_), and after lockdown (t_2_). The highest values are highlighted in each category, and the top three diagnosis in total are also highlighted.

Diagnosis		t_0_ Before Lockdown	t_1_ During Lockdown	t_2_ After Lockdown	Total
gum irritation/wound, gingivitis	N	20	12	35	67
	%	5.2	3.9	7.9	6.0
cleft lips and palates/lip frenulum/oral surgery	N	1	3	1	5
	%	0.3	1.0	0.2	0.4
infection (abscess, fistula, swelling, pus, chronic apical periodontitis)	N	108	66	109	283
	%	28.1	21.2	24.7	24.9
insufficient prothesis/space holder	N	9	26	11	46
	%	2.3	8.4	14.0	11.7
molar incisive hypomineralization	N	6	9	8	23
	%	1.6	2.9	1.8	2.0
poor oral hygiene	N	56	25	67	148
	%	14.5	8.1	15.2	13.0
temporomandibular dysfunction	N	2	1	3	6
	%	0.5	0.3	0.7	0.5
caries/insufficient filling/loss of filling	N	176	119	200	495
	%	45.7	38.3	45.2	43.5
enamel/dentine malformation	N	0	0	1	1
	%	0.0	0.0	0.2	0.1
tooth malposition	N	1	0	4	5
	%	0.3	0.0	0.9	0.4
persisting primary teeth	N	1	0	0	1
	%	0.3	0.0	0.0	0.1
mesiodens	N	2	0	0	2
	%	0.5	0.0	0.0	0.2
postoperative pain	N	16	7	18	41
	%	4.2	2.3	4.1	2.3
pulpitis	N	15	17	11	43
	%	3.9	5.5	2.5	3.6
radix relecita	N	138	158	187	483
	%	35.8	50.8	42.3	42.4

**Table 3:** Treatment procedures among pediatric patients before (t_0_), during (t_1_), and after lockdown (t_2_). The highest values are highlighted in each category, and the top three diagnoses in total are also highlighted.

Treatment Procedures		t_0_ Before Lockdown	t_1_ During Lockdown	t_2_ After Lockdown	Total
prescription of medication	N	106	75	122	303
	%	27.5	24.1	27.6	26.6
wound care (gum)	N	11	5	6	22
	%	2.9	1.6	1.4	1.9
filling	N	17	30	29	76
	%	4.4	9.6	6.6	6.7
endodontic treatment	N	11	13	5	29
	%	2.9	4.2	1.1	2.5
occlusal adaptation, fluoridation, topical appliance of silverdiaminfluoride/tooth mousse	N	4	10	2	16
	%	1.0	3.2	0.5	1.4
extraction	N	53	73	53	179
	%	13.8	23.5	12.0	15.7
no immediate care necessary	N	47	37	70	153
	%	12.2	11.9	15.8	13.4
no therapy due to non-compliance	N	35	31	45	111
	%	9.1	10.0	10.2	9.8
treatment recommendation: general anesthesia	N	45	33	123	201
	%	11.7	10.6	27.8	17.7
appointment for new patient registration	N	146	107	213	466
	%	37.9	34.4	48.2	40.9
referral to further radiological imaging (CT)	N	10	0	0	10
	%	2.6	0.0	0.0	0.9
referral to external dentists/internal referral	N	87	99	129	315
	%	22.6	34.1	29.2	27.7
space holder/prothesis	N	4	22	10	36
	%	1.0	1.6	2.3	3.2

## Data Availability

The original contributions presented in the study are included in the article, further inquiries can be directed to the corresponding author/s.
